# Increased Bone Marrow ^18^F-Choline Uptake in a Patient with Hepatocellular Carcinoma and Thalassemia Intermedia

**DOI:** 10.4274/mirt.galenos.2019.03064

**Published:** 2020-02-17

**Authors:** Luca Filippi

**Affiliations:** 1 Santa Maria Goretti Hospital, Clinic of Nuclear Medicine, Latina, Italy

**Keywords:** 18F-choline, positron emission tomography/computed tomography, hepatocellular carcinoma, thalassemia

## Abstract

A 57-year-old male with history of thalassemia intermedia and hepatocellular carcinoma underwent a positron emission tomography/computed tomography (PET/CT) scan with ^18^F-choline before radioembolization procedure with ^90^Y-microspheres. The PET/CT scan with ^18^F-choline demonstrated highly increased tracer incorporation within a gross lesion in the hepatic dome coupled with diffuse activity in bone marrow, this latter aspect was probably due to the compensatory hematopoiesis stimulation induced by chronic hemolysis. This pattern of skeletal ^18^F-choline uptake should be considered as a peculiar PET/CT finding in thalassemic patients.

## Abstract (tr)

Talasemi intermedia ve hepatosellüler karsinom tanılı bir hastada ^90^Y-mikrosfer ile radyoembolizasyon prosedürü öncesi ^18^F-kolin ile pozitron emisyon tomografisi/bilgisayarlı tomografi (PET/BT) taraması yapıldı. ^18^F-kolin ile PET/BT taraması karaciğer kubbesindeki büyük bir lezyon içerisinde artmış tracer tutulumu ve muhtemelen kronik hemoliz ile indüklenen kompansatuvar hematopoezise kemik iliğinde artmış aktivite gösterdi. Bu skeletal patterned ^18^F-kolin tutulumu talasemik hastalara özgü bir PET/BT bulgusu olarak değerlendirilmelidir.

## Figures and Tables

**Figure 1:**
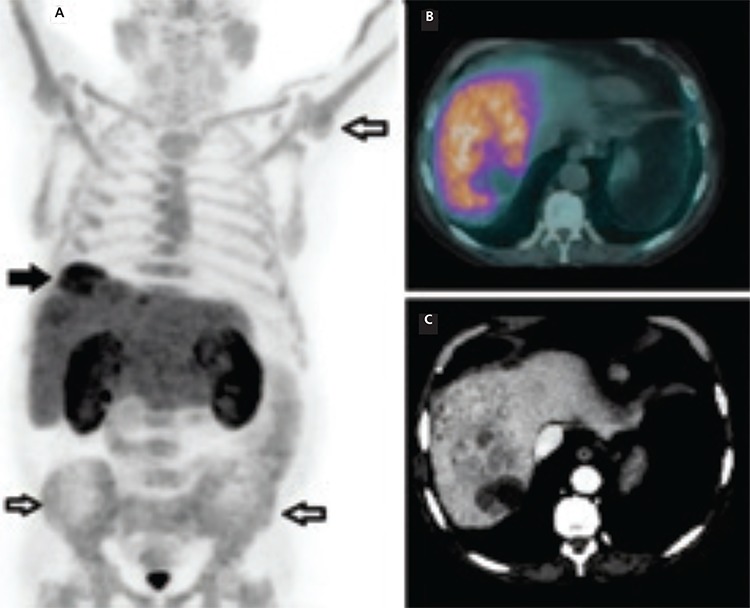
A 57-year-old man was diagnosed as having thalassemia intermedia at the age of 3 years (genotype CD39/IVS 1-6). He received sporadic blood transfusions since childhood and was submitted to splenectomy at the age of 15 years due to giant splenomegaly. Over the years, he developed hemocromatosis secondary to iron overload and was infected by hepatitis C, which was most probably transmitted via blood transfusion before 1990. In April 2018, during a periodical clinical follow-up, an abdominal ultrasound examination revealed multiple lesions in the right hepatic lobe, subsequently confirmed by contrast-enhanced/computed tomography (ce-CT). The patient underwent biopsy which resulted positive for well-differentiated hepatocellular carcinoma (HCC). He received sorafenib until September 2018 when treatment was discontinued due to the onset of cutaneous toxicity and evidence of progressive disease shown by ce-CT. He was enrolled for a loco-regional treatment of the hepatic lesion through radioembolization with ^90^Y-microspheres. Before the radioembolization procedure, he was submitted to positron emission tomography/CT (PET/CT) with ^18^F-choline. **(A)** PET maximum intensity projection showed increased tracer uptake in the hepatic dome (black arrow) and diffuse hyperactivity in the axial and appendicular skeleton (black countered arrows). The corresponding fused PET/CT **(B)** and ce-CT **(C)** axial slices demonstrated multiple lesions, with a necrotic peripheral component, in the right hepatic lobe, characterized by intense ^18^F-choline incorporation with much higher uptake values (SUV_max_: 17.0, SUV_mean_: 6.1) than those calculated in the normal liver parenchyma (SUV_max_: 7.7, SUV_mean_: 5.7).

**Figure 2:**
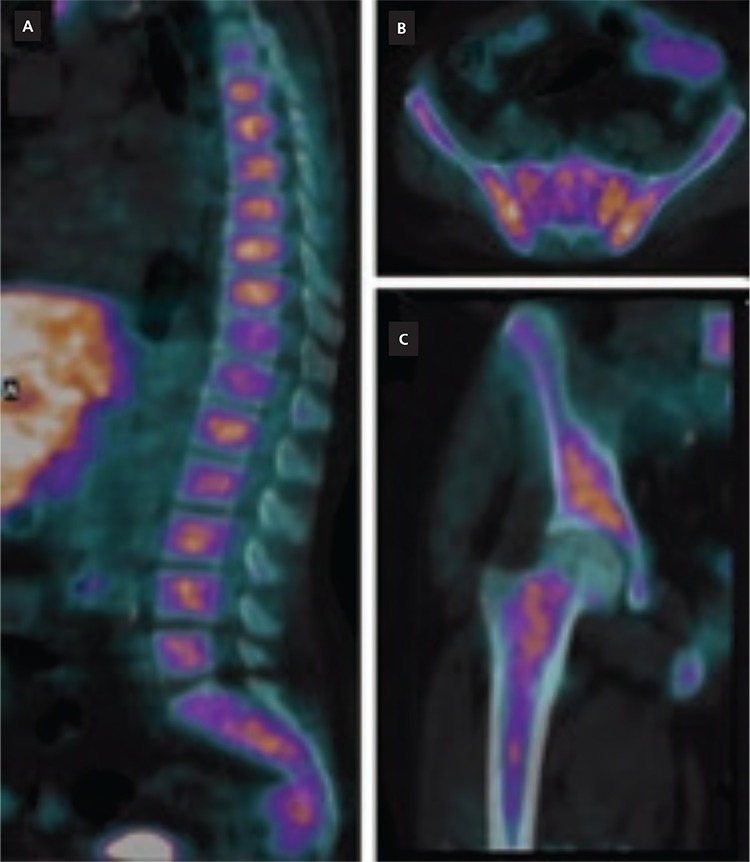
Fused PET/CT well documented tracer incorporation in the endomedullary compartment of the bones, as evident in the sagittal view of vertebrae **(A)**, in the axial slice of the pelvic bone **(B)** and in the detailed coronal view of the right femur **(C)**. Semiquantitative indices measured in bone marrow, specifically in the pelvic bones, showed significantly increased uptake value (SUV_max_: 6.2, SUV_mean_: 4.8) compared with the value reported by Schillaci et al. (1). In a cohort of 80 patients evaluated for assessing the physiological ^18^F-choline biodistribution (i.e. bone marrow SUV_mean_: 2.8). Thalassemia intermedia is a rare inherited genetic disease, characterized by a wide spectrum of clinical manifestations (2). Iron overload due to the chronic hemolysis and periodic blood transfusion leads to severe complications, especially at cardiac and hepatic level. Since recent improvements in treatment of thalassemia have led to a significantly prolonged survival, HCC, most probable related to the frequent association of hepatitis C virusinfection and hemocromatosis in thalassemic patients, has emerged as a relatively new complication in long-term survivors (3). Although conventional radiological imaging through CT and magnetic resonance imaging represents the first-line approach for HCC diagnosis, PET/CT with ^18^F-choline has been introduced as a useful tool for the imaging of HCC, especially before and after loco-regional treatments (4,5). To the best of our knowledge, this is the first report describing the pattern of ^18^F-choline uptake in a thalassemic patient. It has to be pointed out that diffuse skeletal uptake of ^18^F-fluciclovine has been recently described in a thalassemic patient affected by prostate cancer with suspicion of bone metastasis (6). Although ^18^F-fluciclovine and ^18^F-choline represent different molecular probes in oncology, since the former reflects the upregulation of transmembrane aminoacids transport (7) while the latter is a biomarker of phospholid synthesis (8), increased uptake of both these 2 tracers in the bone marrow of thalassemic patients are most probably due to hematopoiesis stimulation induced by chronic hemolysis.
